# The Role of Oocyte Organelles in Determining Developmental Competence

**DOI:** 10.3390/biology6030035

**Published:** 2017-09-18

**Authors:** Karen L. Reader, Jo-Ann L. Stanton, Jennifer L. Juengel

**Affiliations:** 1 Department of Anatomy, University of Otago, PO Box 56, Dunedin 9054, New Zealand; jo.stanton@otago.ac.nz; 2 Animal Reproduction, AgResearch Invermay Agricultural Centre, Private Bag 50034, Mosgiel 9053, New Zealand; jenny.juengel@agresearch.co.nz

**Keywords:** oocyte, oocyte quality, organelles, cytoplasm, oocyte maturation

## Abstract

The ability of an oocyte to undergo successful cytoplasmic and nuclear maturation, fertilization and embryo development is referred to as the oocyte’s quality or developmental competence. Quality is dependent on the accumulation of organelles, metabolites and maternal RNAs during the growth and maturation of the oocyte. Various models of good and poor oocyte quality have been used to understand the essential contributors to developmental success. This review covers the current knowledge of how oocyte organelle quantity, distribution and morphology differ between good and poor quality oocytes. The models of oocyte quality are also described and their usefulness for studying the intrinsic quality of an oocyte discussed. Understanding the key critical features of cytoplasmic organelles and metabolites driving oocyte quality will lead to methods for identifying high quality oocytes and improving oocyte competence, both in vitro and in vivo.

## 1. Introduction

Oocytes vary in their ability to undergo maturation and support embryonic development. This ability is referred to as developmental competence or oocyte quality. An oocyte’s capacity to resume meiosis, complete maturation, undergo successful fertilization, and support normal embryo development is gradually acquired as the oocyte grows [1,2,3,4]. During growth from the primordial follicle stage to ovulation, cytoplasmic organelles replicate and reserves of proteins and RNAs required for post fertilization development are stored [5,6]. Growing oocytes are influenced by factors that regulate follicular growth and by interactions with the surrounding granulosa cells and follicular fluid. While sperm are of course essential for successful fertilization and embryo development, many of the cellular and molecular mechanisms required for fertilization and early embryo development are inherent to the oocyte [6,7]. Therefore, inadequate oocyte growth and maturation adversely affects fertilization and subsequent embryo development. As Swain and Pool [6] wrote: “a prerequisite of obtaining a healthy embryo is first obtaining a healthy oocyte”. Unfortunately, we still lack a clear understanding of the essential features of a good quality oocyte.

In sheep and cattle, between 20% and 40% of embryos are lost during the first two weeks of pregnancy [8,9]. O’Connell et al. [9] reported the majority of early embryo loss in sheep occurs between Days 4 and 14 of gestation (12%), with 6% lost before Day 4 of gestation and minimal losses between Days 14 and 30. This suggests the importance of both oocyte quality and the oviductal environment for embryo survival prior to implantation. Insufficient oocyte quality can also affect post-implantation pregnancy as evidenced by Ptak et al. [10]. These authors monitored pregnancy loss in adult ewes following transfer of blastocysts produced in vitro from adult or pre-pubertal lamb oocytes. Pre-pubertal oocytes are known to have poor developmental competence [11]. While 60% of embryos transferred from lambs survived at Day 40 of gestation, there was significant loss between Days 40 and 60 of gestation with further loss before term [10]. There was no significant pregnancy loss from embryos transferred from adult ewes after Day 40 of gestation.

Assisted reproductive technologies (ART) are widely used to overcome infertility in all animals including humans. ART techniques such as in vitro production of embryos (IVP) with cryopreservation and embryo transfer, offer the ability to increase the rate of genetic gain in farmed animals [12] and to conserve endangered species. Progeny can be produced from high genetic value females by oocyte collection and IVP, in situations such as prepuberty, pregnancy or after death. Juvenile in vitro embryo transfer (JIVET) can decrease the generation interval and increase the rate of genetic gain for livestock-improvement by producing embryos and offspring from juvenile animals [13].

Despite ART being widely used, success rates are still low. For example, the live birth rate from an average of 1.6 embryos transferred per women under the age of 35 years is 43.4%, per egg-retrieval cycle, as reported by the Society for Assisted Reproductive Technologies in 2014 (SART; www.sart.org). The rate of implantation steadily declines with age. Therefore, even for the younger age group with good pregnancy prognosis, the majority of the embryos transferred will not implant [14]. Diseases such as cancer, autoimmune or hematological conditions often require chemotherapy or radiotherapy treatment that can result in premature ovarian failure [15]. Relatively new techniques, such as the cryopreservation of oocytes or ovarian tissue, are being developed and used to retain fertility in these patients. However, the live birth rate per vitrified mature oocyte is currently only 5.9% [16]. Cryopreservation and in vitro maturation of immature human oocytes is even less successful [17].

In cattle, around 20–30% of oocytes retrieved and fertilized in vitro develop into transferable embryos, with 50% of the embryos transferred resulting in pregnancy and 5–10% of these lost before birth [18]. Success rates in sheep and goats appear similar to that observed in cattle [19]. Hence, across the board improvements in the selection of good quality oocytes and embryos, and culture systems that can promote these are required.

Oocytes must undergo correct nuclear and cytoplasmic maturation following the luteinizing hormone (LH) surge. Nuclear maturation is dependent on the formation and maintenance of the meiotic spindle which requires centrosomal proteins, such as the nuclear mitotic apparatus and γ-tubulin, as well as other regulatory proteins. Oocyte aging is known to cause dissociation of these centrosomal proteins and disintegration of the meiotic spindle, resulting in aneuploidy and decreased developmental potential. Several recent reviews on nuclear maturation and embryo development have been published [20,21]. Cytoplasmic maturation involves the reorganization of organelles and the final storage of mRNAs, proteins, lipids and transcription factors required during fertilization and early embryogenesis [3,6,22,23,24]. This has also been termed oocyte capacitation; the ability of the oocyte to successfully accomplish the cytoplasmic aspects of maturation [1]. It is well known that even if oocytes are able to undergo successful nuclear, or meiotic, maturation to the metaphase II stage (MII) of meiosis II, when the first polar body is extruded, the oocytes may not have accomplished adequate cytoplasmic maturation to complete embryo development [3].

Markers of successful cytoplasmic maturation are not well defined or easily visualized making it difficult to assess oocyte cytoplasmic quality [6]. Several oocyte controlled molecular and cellular events that must occur to allow effective fertilization have been described by Swain and Pool [6], and these include; zona pellucida formation, cumulus cell expansion, calcium storage and signaling, cortical granule relocation and release, sperm processing and pro-nuclei formation. Oocyte cytoplasmic organelles such as mitochondria, lipid droplets and cortical granules play a key role in determining oocyte quality. The involvement of these organelles also provides information about metabolic pathways important to the oocyte.

Increasing our knowledge of which organelles, metabolites and metabolic pathways are important for determining oocyte competence will allow potential improvements to the success of both natural fertility and ART. A recent review by Labrecque and Sirard [25] provides an overview of the current knowledge of transcriptomic differences between oocytes of different quality. These authors report that a clear set of expressed genes that determine oocyte competence is yet to be identified. This may be due to the poor correlation between mRNA and protein expression in oocytes, and high variability between oocytes. Comparing the quantity and quality of organelles and metabolites between oocytes of differing quality could provide a better understanding of factors regulating oocyte competence and identify ways that oocyte quality can be manipulated. This requires the use of good models of high and low oocyte developmental potential along with accurate methods for quantifying oocyte components. This review will describe current models of oocyte competence and discuss the appropriateness of each model for studying inherent oocyte quality. It will focus on quantitative studies of organelles involved in cytoplasmic oocyte maturation. We will then explore current knowledge of the relationship between organelle quantity and distribution, and oocyte developmental potential. Finally, we will discuss how this knowledge could lead to improvements in both in vitro and in vivo oocyte quality.

## 2. Models of Oocyte Competence

Many different models of high or low oocyte developmental competence have been used to study factors important for oocyte quality. To be effective these experimental models must compare high and low quality oocytes under similar environmental conditions and be able to clearly distinguish differences in features inherent to these oocytes. Some commonly used models for examining intrinsic organelle differences in relation to oocyte quality will be described in this section.

### 2.1. Follicle and Oocyte Size

Oocytes from larger follicles are generally considered to have higher developmental potential than those from smaller follicles as it is hypothesized that they have had more time to grow and accumulate the necessary components for maturation [2,26,27,28,29]. In this view, the difference in oocyte competence depends on whether follicle size accurately indicates oocytes that have completed growth. Fair et al. [30] reported a small positive correlation (r = 0.32) between follicular size and oocyte diameter but also found some fully-grown bovine oocytes in smaller follicles. Ptak et al. [10] also found a positive correlation between follicle and oocyte diameter in adult ewes (r = 0.61) but this correlation was absent in prepubertal lamb ovaries (r = 0.11). Thus, follicle size may not be a reliable predictor of oocyte quality and the size of the oocyte itself may be a more useful indicator.

Pig oocytes with a larger volume have been shown to have higher fertilization rates than smaller oocytes [31]. Accordingly, in humans, embryos from the pregnant and younger age groups started with larger oocyte volumes when compared to unfertilized oocytes or uncleaved zygotes [32]. Human embryos undergoing earlier cleavage also had larger volumes than those developing more slowly. Reader et al. [33] and Gandolfi et al. [34] reported oocytes from adult sheep and cows respectively, presumably with higher developmental competence, were larger than oocytes from their juvenile counterparts. In contrast, O’Brien et al. [35] and O’Brien et al. [36] found no difference in the size of oocytes harvested from juvenile or adult pigs or sheep. Adult ewe and lamb oocytes reach their maximum diameter in follicles that are between 0.4 and 0.6 mm in diameter [37,38]. Thus, lamb and adult ewe oocytes that were aspirated from follicles greater than 1 mm diameter [33] or between 2 and 4 mm diameter [35] were expected to be fully grown.

Conflicting observations from some of these studies could be due to the methods used for determining oocyte size and the number of oocytes measured. Oocyte diameter can be measured non-destructively but, as oocytes are not perfectly spherical, more than one measurement should be made. The square root of the maximum diameter multiplied by the diameter at right angles estimates true diameter, better than a single measurement [39]. Most of the papers referenced above calculated the oocyte volume (either total or cytoplasmic) from the diameter only. However, this does not account for the irregular shape of the oocyte ooplasm and the size of the perivitilline space. The only methods available for measuring volume accurately rely on stereological techniques or confocal microscopy and thus are destructive. Cytoplasmic volume may better reflect the amount of organelles and accumulation of metabolites that influence oocyte quality rather than total oocyte diameter that includes the zona pellucida, which can vary in thickness. Overall, it appears that larger oocytes are of better quality.

### 2.2. Glucose-6-Phosphate Dehydrogenase Activity

A popular method for identifying competent oocytes relies on staining with brilliant cresyl blue (BCB) to measure glucose-6-phosphate dehydrogenase (G6PD) activity. BCB is promoted as a non-toxic method for identifying oocyte potential. The activity of G6PD decreases in oocytes that have completed growth [31]. These oocytes are unable to reduce BCB to a colorless compound and therefore retain the blue dye (BCB+). Oocytes that are still growing appear unstained (BCB−). A higher proportion of BCB+ oocytes develop to blastocyst stage than BCB− oocytes [40,41,42]. Nonetheless, not all BCB+, and some BCB−, oocytes are competent. This technique is based on a single metabolic pathway and differences in competence are likely to be due to differences in the stage of oocyte growth rather than reflecting intrinsic differences in oocyte quality [43].

### 2.3. In Vivo Versus In Vitro Maturation

Oocytes matured in vitro are less developmentally competent than those matured in vivo [3,44] and both systems have been used to compare oocyte quality. For example, Rizos et al. [44] compared blastocyst rates between immature bovine oocytes that were in vitro matured and fertilized, and in vivo matured oocytes collected following synchronization and superovulation. In vivo matured oocytes had higher blastocyst rates than those matured in vitro.

The differences observed between in vitro and in vivo matured oocytes are most likely induced by the differences in the maturation environment rather than due to fundamental oocyte quality. This makes it difficult to identify the factors responsible for altering oocyte competence [25]. Good quality oocytes may be able to tolerate less optimal conditions than poorer quality oocytes but this is difficult to prove, as the quality of the oocyte is not usually known before the experiment. This model is however useful for determining ways of improving in vitro embryo production methods. It may also provide insights into which oocyte genes, organelles and metabolic pathways are affected by a suboptimal maturation environment, and are therefore important for oocyte quality.

### 2.4. Maternal Age

There is clear evidence that in adult animals, including humans, oocyte competence reduces with increasing age [45,46]. Much of the current literature also agrees that oocytes from prepubertal mammals; including rodents, cattle, sheep and pigs; have limited potential to undergo normal embryogenesis and produce viable offspring when compared to their adult counterparts, as reviewed by Armstrong [11].

The blastocyst rate is decreased in embryos produced in vitro from prepubertal sheep, cow, pig and mouse oocytes compared to those derived from adults [47,48,49,50,51,52,53]. In two similar studies, only 0.7% and 4% of harvested lamb oocytes produced offspring compared to 13% and 11% of oocytes from adult ewes following in vitro embryo production (IVP) and transfer to adult recipients [10,48]. Similarly, in the cow, calving rates from IVP embryos transferred to adult recipients were 22% for embryos produced from calf oocytes and 39% for cow embryos [54]. Therefore, both juvenile and aged oocytes provide a good model for studying the mechanisms that underlie acquisition of developmental competence [55,56].

Methods for improving oocyte quality in ART may be developed through understanding what limits oocyte competence in these maternal age models. Consequently, women delaying reproduction or undergoing early onset menopause may still be able to reproduce and JIVET may become a more efficient method for increasing the rate of genetic gain in farmed species.

Models described in the section above have been used in studies to identify factors contributing to developmental competence. The following sections of this review focus on research that has measured organelle quantity differences between good and poor oocytes during maturation.

## 3. Mitochondria and Oocyte Quality

Mitochondria are the most commonly studied organelle relative to developmental competence. Their essential roles in the oocyte include ATP production and the control of Ca^2+^ and redox homeostasis, as reviewed by Dumollard et al. [57]. Several mitochondrial parameters appear altered in oocytes with high or low developmental competence, with improved oocyte quality attributed to an increased number of mitochondria, altered cytoplasmic mitochondrial distribution and higher mitochondrial membrane potential [31,33,58,59].

### 3.1. Mitochondrial Morphology

**Figure 1:**
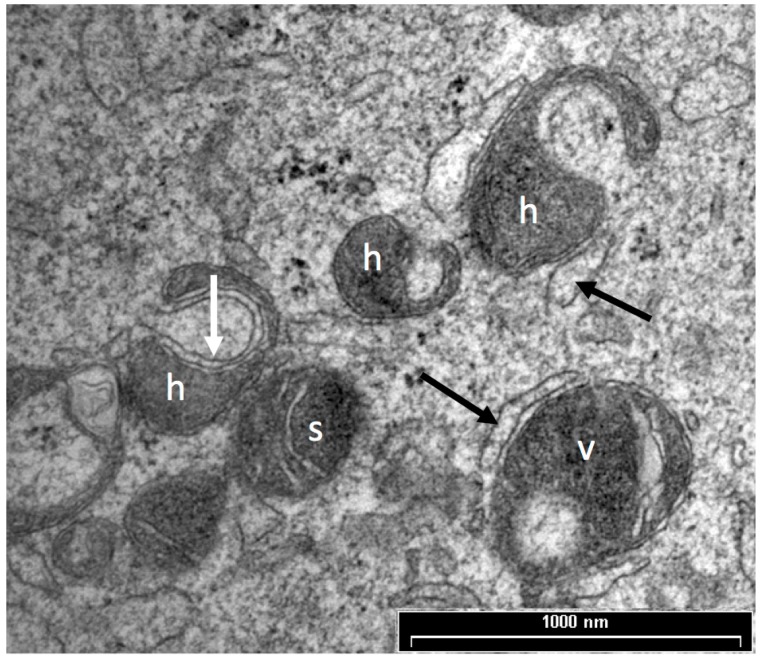
TEM image of spherical (s), hooded (h) and vacuolated (v) mitochondria in an in vitro matured adult sheep oocyte. Smooth endoplasmic reticulum can be observed closely associated with the outer mitochondrial membrane (black arrows) and within the hood of the mitochondria (white arrow). Scale bar = 1 μm.

**Figure 2:**
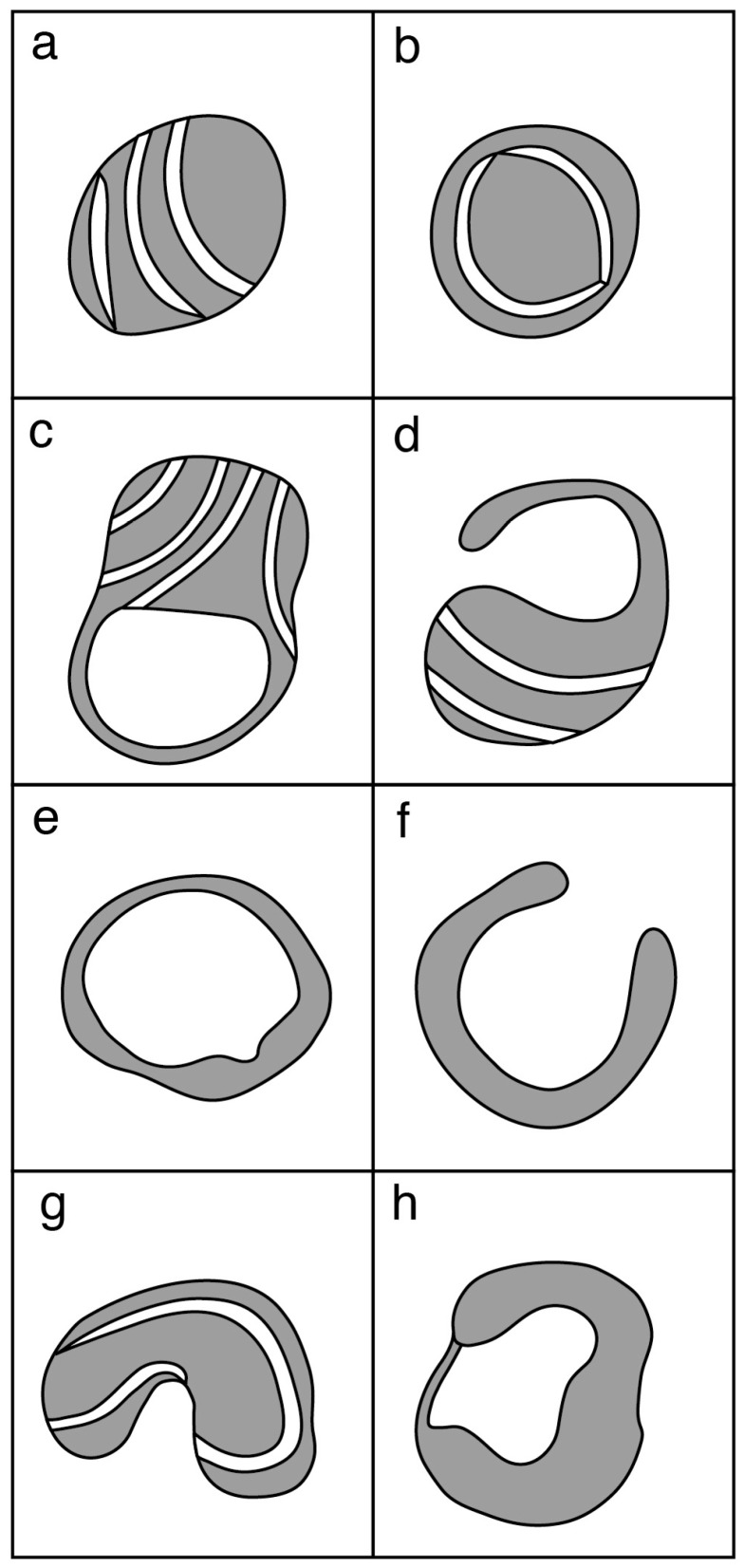
Diagram of the profiles of mitochondria observed in TEM images of oocytes from the human, cow, sheep, pig and mouse: (**a**) typical spherical with transverse cristae; (**b**) spherical with peripheral cristae; (**c**) vacuolated; (**d**) hooded or cap-shaped; (**e**) ring-shaped; (**f**) cup- or horseshoe-shaped; (**g**) elongated, irregular; and (**h**) shell-like. Profile (**a**) has been observed in oocytes from all these species; (**b**) in all except sheep; (**c**) in cow, sheep and mouse oocytes; (**d**) in human, cow and sheep oocytes; (**e**) in cow and mouse; (**f**) in human and sheep; (**g**) in pig; and (**h**) in sheep, pig and mouse. See text for references.

Prior to oocyte maturation the mitochondria are mainly spherical with few cristae and are assumed to be relatively inactive. Oocytes from some species, including cow, sheep, and goat, have a proportion of mitochondria with an arc-like structure or “hood” that have been described as hooded or cap-shaped mitochondria [5,38,60]. In cow and sheep oocytes, the hooded form of mitochondria (Figure 1) becomes more prevalent during the later stages of growth and maturation [5,33,38,61]. In human oocytes, hooded, ring- or horseshoe-shaped mitochondria have been observed [62,63]. Mouse oocytes contain numerous vacuolated or ring-like mitochondria that appear similar to hooded mitochondria except that the space within the organelle is shown to be completely enclosed in all of the observed sectioned mitochondria [64,65,66]. Hooded mitochondria are rarely seen in pig oocytes [67,68]. Diagrams of the various forms of mitochondria observed in mature oocytes from human, cow, sheep, pig and mouse are shown in Figure 2.

Changes in mitochondrial morphology are seen in several models of oocyte quality based on maternal age. Simsek-Duran et al. [65] showed differences in the ultrastructure of mitochondria between oocytes from young and old mice and hamsters. Older mice, which have reduced fertility, had a higher percentage of vacuolated mitochondria in their oocytes than younger animals. Aged hamsters had a higher proportion of mitochondria with electron dense matrices and less with clearly visible cristae compared to the oocytes from young, more fertile, animals. Juvenile lamb oocytes had a higher proportion of hooded mitochondria before maturation, and a lower proportion after maturation, compared to the more competent adult ewe oocytes [33]. In the pig, oocytes from prepubertal animals had mitochondria that were compartmentalized and contained granules, while the more competent, post-pubertal oocytes were described as shell-like with no granules [68]. Thus, in general, poor quality oocytes were characterized by altered mitochondrial morphology including denser matrices, the presence of granules or vacuoles and an altered timing for the formation of hooded mitochondria.

The functional significance of these mitochondrial morphological differences is unclear but the morphology may reflect changes to the energetic state of the mitochondria as reviewed by Galloway et al. [69]. Inhibitors of components of the electron transport chain, chemical uncoupling with carbonyl cyanide phenylhydrazones (FCCP, CCCP), and hypoxic conditions all induce the formation of donut-shaped mitochondria in various cell types [70,71,72,73,74]. However, there does not appear to be similar published studies that relate changes in mitochondrial morphology to function in oocytes. Such research could potentially provide insight into improving mitochondrial function in oocytes.

### 3.2. Mitochondrial Distribution

In most species mitochondria move from a peripheral location to a more even distribution throughout the oocyte cytoplasm during maturation [33,38,67,75,76]. In the mouse GV stage oocyte, however, mitochondria are evenly distributed and migrate to a perinuclear location during germinal vesicle breakdown (GVBD) [64,77]. In mouse oocytes, abstriction of the polar body is accompanied by movement of mitochondria with the nucleus to the periphery of the cell. Following abstriction, the mitochondria become evenly distributed again. This suggests the mitochondria are providing a local supply of ATP for GVBD and relocation of the chromosomes [64]. Mouse oocytes that remain at GV stage or fail to mature show no mitochondrial translocation. Perinuclear distribution of mitochondria associated with polar body abstriction may not feature in pig, cow and sheep oocytes maturation because the nucleus in these species tends to be already located peripherally.

The distribution pattern of oocyte mitochondria has been linked to developmental competence in several species including the mouse, cow, pig and sheep [33,35,78,79,80]. Mature oocytes from cows and pigs with greater developmental potential were more likely to have mitochondria distributed evenly throughout the cytoplasm than their poorer quality cohorts [79,80]. In sheep, O’Brien et al. [35] reported a greater density of mitochondria in the cortex of adult animals after IVM, compared to the middle of the oocyte; while in prepubertal lamb oocytes, the density was the same in both regions. In contrast, Reader et al. [33] showed that prior to maturation, less competent prepubertal lamb oocytes have a higher density of mitochondria in the center but mitochondria become evenly distributed in both adult and lamb oocytes after maturation. Similarly, Machatkova et al. [81] reported that bovine oocytes from small and medium follicles both had evenly distributed mitochondria after IVM. They also reported a higher percentage of the immature oocytes from small follicles had peripherally located mitochondria than those from the medium sized follicles.

In general, higher oocyte quality is associated with an even distribution of mitochondria throughout the cytoplasm of the mature oocyte. Since mitochondria localize to areas where either high levels of ATP or Ca^2+^ signaling are required [82], the relocation of mitochondria during the maturation process may be crucial to oocyte function. In addition, an even distribution of mitochondria is believed to be important prior to cleavage, to ensure that each blastomere receives sufficient mitochondria to survive early embryogenesis [83].

### 3.3. Mitochondrial Quantity

Various methods have been developed to measure mitochondrial quantity. These include morphometric analysis of organelle number, quantification of mtDNA copy number and measurements of mitochondrial activity. While these methods all focus on measuring mitochondria, they each have strengths and limitations when applied to oocyte quality research.

Morphometric, or stereological, analysis using electron microscopy can provide information regarding density, morphology and size of the mitochondria, but while morphology may provide some information on mitochondrial activity, this is not well understood. It is a time consuming, complex procedure that requires specialist skills and equipment thus limiting the number of samples that can be practically assessed. Stereological methods for quantifying organelles require robust random sampling to prevent bias due to uneven organelle distribution. In contrast, measurement of mtDNA copy number by quantitative PCR is relatively simple to undertake in a well-equipped molecular biology laboratory. Thus, this method is amenable to obtaining data from a large number of samples. However, the relationship between mtDNA copy number, the number of mitochondria and mitochondrial activity has not been clearly established in oocytes. The actual number of mtDNA copies per organelle is difficult to confirm unequivocally [84,85]. It is assumed that there are between one and two copies of the mitochondrial genome per mitochondria but this appears to vary, with one study suggesting greater than two genome copies per mitochondria [59] while another indicated less than one genome copy [33]. In addition, there is greater variation in mtDNA copy number among oocytes when compared to mitochondrial number measured by stereology [33,86].

Quantification of mitochondria has also been measured with MitoTracker dyes, which are selectively concentrated in active mitochondria. MitoTracker Green is taken up by active mitochondria but is relatively insensitive to mitochondrial membrane potential (MMP) [87] and therefore has been used as a means to quantify active mitochondrial mass or number. Inactive mitochondria are not stained by MitoTracker Green and, in the case of the oocyte which is thought to contain a high proportion of mitochondria that are relatively inactive, MitoTracker Green fluorescence intensity may not provide a very accurate estimation of overall mitochondrial mass. Other mitochondrial specific fluorescent dyes, such as MitoTracker Red, MitoTracker Orange and JC-1 are affected by MMP. This means that the intensity of fluorescence is dependent on the activity levels of the mitochondria. Thus, these dyes measure mitochondrial activity but they are not a good method for measuring overall mitochondrial mass. An important aspect of MitoTracker Orange is that under specific conditions it inhibits respiratory complex I and induces mitochondrial permeability potentially making it unsuitable for measuring changes in MMP [87].

Making quantitative comparisons using fluorescent dyes and microscopy requires careful control for technical differences between individual samples such as stain uptake, oocyte thickness, light scattering and absorbance by the specimen. The microscope settings must also be standardized between individual samples. In confocal microscopy the power density of the illuminating spot decreases as the depth of the specimen increases and hence the amount of light emitted from the fluorophore will diminish with depth [88]. Thus, signal will vary across the diameter of a spherical oocyte as a function of cell depth not mitochondrial number. This means mitochondrial-specific dyes are useful for measuring mitochondrial activity only when used under well-defined conditions. The limitations of the method used to quantify mitochondria need to be considered when interpreting the results.

#### 3.3.1. Mitochondrial Organelle Number and Oocyte Quality

Replication of mitochondria has been shown to occur during oocyte growth but not during cleavage [85,89,90]. Consequently, the presence of an adequate number of evenly distributed mitochondria in the mature oocyte is believed to be essential to supply the energy requirements of each blastomere during early embryo development [57]. Studies in humans report the birth of healthy babies from patients with recurring implantation failure following ooplasmic transfer from younger donors. The successful pregnancies were believed to be due to transfer of functional and/or additional mitochondria to the oocytes [91,92] but no controlled studies have been performed to prove this and the practice has been discontinued in some countries due to concerns about biological safety and the ethics of generating offspring with genetic material from three parents.

Mitochondrial density, volume and number were measured in oocytes from prepubertal lamb and adult sheep using electron microscopy and stereological techniques [33]. The total volume and number of mitochondria per oocyte increased in adult ewes during maturation but not in lamb oocytes with the overall mitochondrial volume and number being greater in adult oocytes compared to the lamb oocytes after IVM. Lamb oocyte mitochondria were larger than those in adult oocytes after maturation. Two other quantitative ultrastructural studies also reported a greater volume fraction (V_V_), or density, of mitochondria in adult cow and sheep oocytes compared to prepubertal oocytes following maturation but the numerical density (N_V_) of mitochondria did not differ [35,93]. These results indicate that the adult oocyte mitochondria were larger which differs from the results of Reader et al. [33] in the sheep. Pedersen et al. [68] counted mitochondria in similar sized, immature oocytes from pre- and post-pubertal pigs that were four to six months old. This study found no difference in the number, volume or density of mitochondria between these two groups of oocytes, however, smaller prepubertal oocytes had fewer mitochondria but at a similar density. The small number of studies that have compared mitochondrial organelle number between oocytes of differing quality generally support the premise that better quality oocytes have more mitochondria.

#### 3.3.2. Mitochondrial DNA Copy Number and Oocyte Quality

Numerous studies across different species found no difference in the number of copies of mitochondrial DNA in fully-grown oocytes before and after maturation [94,95,96,97,98]. Conversely, studies by Pawlak et al. [99] in pig and Iwata et al. [46] in cow showed a significant increase in mtDNA copy number in oocytes after maturation. All studies reported high inter-oocyte variation in mtDNA copy number. The Pawlak study examined a much larger sample size than the other reports, which may have enabled statistical differences to be observed between the immature and mature oocytes. 

The average mtDNA copy number in both human and pig oocytes was significantly lower in cohorts that failed to fertilize compared to cohorts with normal fertilization [31,100]. Mitochondrial DNA copy number was also reported to be lower in oocytes from older woman, and from aged mice and hamsters compared with younger, more fertile, animals [32,65]. There appeared to be trend toward a gradual decline in mtDNA copy number with age in cows from 50 months of age and older [46]. However, there was no relationship between mtDNA copy number and maternal age in oocytes from young (21 to 89 months) and old cows (≥90 months) despite the older cows having an increased rate of abnormal fertilization. Similarly, there was no difference in the mtDNA copy number between prepubertal and adult ooctyes in sheep and pigs [33,99,101].

Embryos that cleaved at a faster rate, and are presumed to be of better quality, had a larger oocyte volume with a positive correlation indicated between blastomere volume and mtDNA copy number in human embryos [32]. Several other models of oocyte quality have also been reported to have greater mtDNA copy numbers in oocytes with higher developmental potential including; rat oocytes from larger follicles [102]; mouse oocytes following a natural cycle as opposed to controlled ovarian hyperstimulation or IVM [103]; and BCB+ oocytes from pigs [31]. Ge et al. [104] have also shown that reducing mtDNA copy number in mouse oocytes decreases blastocyst rate.

Overall current evidence supports the idea that highly competent oocytes have a greater mtDNA copy number, than less competent oocytes.

#### 3.3.3. Mitochondrial Activity and Oocyte Quality

The degree of mitochondrial activity and levels of ATP production in bovine oocytes have been shown to increase during IVM [58,79,81,105]. However, contradictory results from studies comparing the intensity of mitochondrial specific fluorescent dyes, such as MitoTracker Red or MitoTracker Orange, between oocytes positive or negative for BCB staining are reported. Torner et al. [41] reported lower mitochondrial fluorescence in immature bovine BCB+ oocytes compared to BCB− oocytes, while Castaneda et al. [106] found no differences between BCB+ and BCB− immature bovine oocytes. Catala et al. [42] also reported no difference in mitochondrial fluorescent intensity before maturation between BCB+ and BCB− prepubertal sheep oocytes, but mitochondrial activity decreased in BCB− oocytes after IVM. These contradictory observations may be due to different culture media, oxygen tension or the mitochondrial stains used.

Bovine oocytes with dark cytoplasm had greater developmental potential than pale or brown oocytes, and also had greater fluorescent intensity when stained with MitoTracker Green than the paler oocytes [107]. Machatkova et al. [81] found that bovine oocytes from small (2 to 5 mm) follicles exhibited an increase in the intensity of MitoTracker Orange during maturation while there was no change to the intensity of oocytes from medium follicles (6 to 10 mm). ATP production increased during maturation in the oocytes from both medium and small follicles. ATP content was also higher in “category 1” oocytes, graded on their morphology and exhibiting higher blastocyst rates, than those from “category 3 and 4” [79]. Both mitochondrial activity, measured with JC-1 stain, and ATP levels were higher in mouse oocytes collected following either a natural cycle or IVM when compared to those from controlled ovarian hyperstimulation treatment [103]. While IVM oocytes had similar levels of mitochondrial activity and ATP to oocytes from natural cycles, they exhibited higher levels of potentially damaging reactive oxygen species (ROS).

Ge et al. [104] also reported inhibition of oxidative phosphorylation in mouse oocytes reduced MMP, ATP, and re-distribution of mitochondria as well as maturation and blastocyst rates showing that, as expected, mitochondrial activity is important for oocyte quality. However, too much mitochondrial activity may produce increased levels of ROS and thus be detrimental to oocyte quality [108]. Reducing mtDNA copy number with 2’,3’-dideoxycytidine in mouse oocytes had no effect on MMP, ATP levels, mitochondrial distribution or oocyte maturation and fertilization [104]. It did however decrease blastocyst rate. These authors concluded that mitochondrial activity and ATP production did not correspond to mtDNA copy number.

It is difficult to conclude, from the studies described in this section, whether increased mitochondrial activity corresponds to increased or decreased oocyte quality. Changes in ATP levels occur in seconds and can be due to alterations in production and/or consumption which are perhaps more likely to correspond to variations in the immediate environment rather than to the intrinsic quality of the oocyte. It is therefore the ability of the oocyte to respond to environmental perturbations that denotes its quality.

### 3.4. Summary of Mitochondria in Oocyte Quality

As a whole, studies examining differences in mitochondria in relation to developmental competence provide evidence that a critical number, distribution pattern and morphology of mitochondria are required for successful oocyte and embryo development. The relationship between mtDNA copy number, mitochondrial number, ATP production and MMP are still unclear and need further exploration.

## 4. Role of Lipid Droplets in Oocyte Quality

It is well known that mammalian oocytes contain large stores of lipid and that the relative abundance of lipid is species specific. Ultrastructural studies have demonstrated high numbers of lipid droplets in the cytoplasm of bovine, porcine and ovine oocytes [67,75,109]. However, mouse oocytes have smaller and fewer electron-dense lipid droplets than domestic animal species while human oocytes appear to have none. The essential role played by fatty acids in oocyte developmental competence has been recently reviewed by Dunning et al. [110]. Very few studies have examined the composition and function of the oocyte lipid reserve [111,112]. McEvoy et al. [111] showed the fatty acid content of pig oocytes was around two-fold that of cattle and sheep oocytes. The three most prevalent fatty acids in these three species were palmitic, oleic and stearic acid which together make up approximately two thirds of the total fatty acid mass. Saturated fatty acids are more abundant than mono- or poly-unsaturated fats. Oocyte fatty acids are believed to be a source of metabolite for energy production, demonstrated by the inhibition of β-oxidation during oocyte maturation which led to decreased embryo viability in the pig, cow and mouse [113,114,115]. Oocytes also contain phospholipids and cholesterol that are important for the formation of membranes required for repeated cell division to form an embryo. Many cell signaling molecules are also formed from fatty acid precursors [111,116,117].

### 4.1. Lipid Droplet Quantity

Crocomo et al. [75] described an apparent reduction in the number of lipid droplets in sheep oocytes after IVM while Reader et al. [33] reported no difference in the volume of lipid droplets before and after IVM. Results from lipid measurements in cow oocytes are also contradictory. When stained with Nile red the intensity of fluorescence in cow oocytes decreased after IVM and there appeared to be less lipid droplets in ultrathin sections, indicating a decrease in total lipid content [118]. However, using Bodipy stain and confocal microscopy Warzych et al. [119] reported an increase in lipid droplet number in oocytes from adult cows after IVM. Quantitative ultrastructural studies of pig oocytes showed the number of lipid droplets per area, or numerical density (N_V_), increased towards the end of maturation, although the volume density (V_V_) did not change [67]. Quantitative ultrastructural studies comparing oocytes from juvenile and adult cows and sheep found no difference in the volume density, numerical density or total volume of lipid droplets between the age groups [33,93]. Warzych et al. [119], however, reported an increase in lipid droplet number in adult cow oocytes during IVM but no change in prepubertal heifer oocytes.

Removal of cumulus cells prior to IVM reduces oocyte developmental competence (see Tanghe et al. [120] for review) and Auclair et al. [118] have reported a reduction in total lipids as revealed by Nile red staining in denuded oocytes as compared to cumulus enclosed oocytes. This did not correspond to significant differences in the area fraction of lipid droplets between immature oocytes, mature denuded oocytes and cumulus enclosed oocytes. This agrees with studies in the cow and sheep [33,93]. The electron microscopy studies from de Paz et al. [93] and Reader et al. [33] quantified lipid droplets stained with osmium tetroxide which binds mainly to unsaturated lipids [121]. Nile red stains both saturated and unsaturated fats and may be more appropriate for quantifying total lipid [122]. Differences between studies may also be due to differing concentrations of lipids present in the media. This would influence whether the oocyte utilized its own lipid stores or could incorporate fatty acids from the environment.

Oocytes with darker cytoplasm are believed to contain more lipid droplets and have better developmental outcomes than paler oocytes [107]. Darker oocytes contain more saturated stearic acid than lighter colored oocytes that have higher amounts of oleic and linoleic fatty acids although the amounts of saturated palmitic acid are similar in both [123]. Therefore, oocyte quality may actually depend on the amount of a particular type of lipid rather than total lipid quantity. Aardema et al. [124] showed that both palmitic and stearic acid reduced bovine oocyte competence when added during IVM, while oleic acid improved development and could counteract the negative effects of palmitic and stearic acid. Oocytes that stain positively for BCB, indicating reduced G6PD activity, have high competence compared to BCB negative oocytes. Castaneda et al. [106] reported that BCB positive bovine oocytes had greater amounts of lipid stained with a Bodipy neutral lipid fluorescent probe than BCB negative oocytes.

### 4.2. Lipid Droplet Distribution

**Figure 3:**
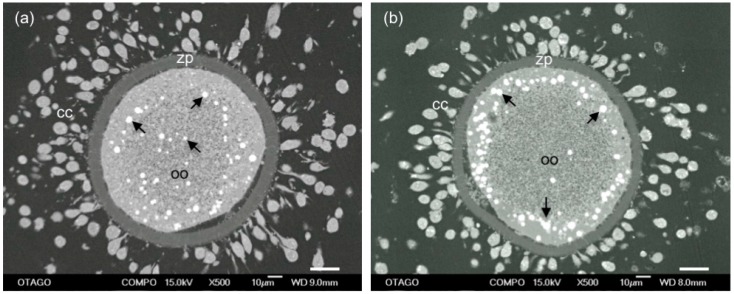
SEM images of lipid distribution in mature sheep oocytes (oo) surrounded by zona pellucida (zp) and expanded cumulus cells (cc). Lipid droplets (arrows) are either distributed: throughout the cytoplasm (**a**); or in a peripheral location (**b**). A higher proportion of oocytes from adult sheep had evenly distributed lipid droplets than those from prepubertal sheep that had predominantly peripherally located droplets. Scale bar = 20 μm.

As with mitochondria, lipid droplet distribution changes during oocyte maturation and differs between species. Lipid droplets were located peripherally in pig oocytes, centrally in mouse oocytes and evenly in sheep and cow after in vitro or in vivo maturation [119,125,126,127]. Warzych et al. [119] demonstrated that lipid droplets were located peripherally in cow oocytes before IVM and became evenly distributed after IVM. While prepubertal and adult cow oocytes were examined in this study, differences in lipid distribution between the two age groups were not described. Comparison between adult ewe oocytes and prepubertal lamb oocytes after IVM showed lipid droplets were evenly distributed in a higher proportion of adults oocytes, with a predominantly peripheral distribution in oocytes from lambs [127]. Scanning electron microscopy images of peripherally and evenly distributed lipid droplets in sheep oocytes are shown in Figure 3. In pigs, however, the lipid droplets were evenly distributed in prepubertal oocytes and centrally located in postpubertal oocytes both before and after IVM [68]. Therefore, lipid droplet distribution may be important for oocyte quality but varies between species. Since fatty acids have been shown to be an essential source of energy in oocytes [110], it may be important for the mitochondria and lipid droplets to be co-located to facilitate the transport of fatty acids into the mitochondria for beta-oxidation. Further research is needed to understand how lipid droplet number, size and location influence oocyte quality.

## 5. Vesicles and Oocyte Quality

**Figure 4:**
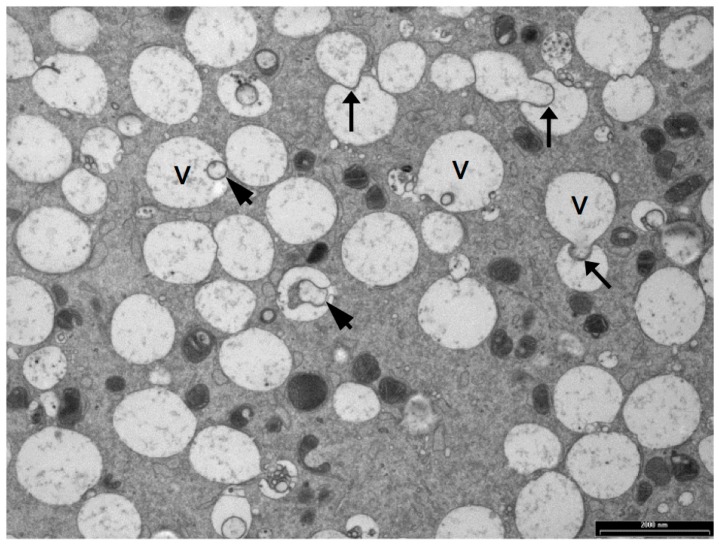
TEM image of the cytoplasm of a sheep oocyte with vesicles (v) containing granular material or membranous structures (arrowheads), and appearing to bud or coalesce with other vesicles (black arrows). Scale bar = 2 μm.

Oocyte vesicles have been described in a large number of species including human, sheep, cattle, pigs, mice and possums [38,62,67,76,128,129,130]. When oocytes are prepared by conventional TEM preparation methods, the vesicles appear as large, translucent organelles, approximately 1–3 µm in diameter, sometimes with a distorted or interrupted membrane. Some vesicles contain amorphous and membranous material and are often closely associated with endoplasmic reticulum. Figure 4 shows a transmission electron microscope image of vesicles in a sheep oocyte. Despite the fact that they occupy between 15% and 36% of the oocyte cytoplasm [33,35,67] the contents and functions of these organelles have not been identified [75]. There is some evidence that oocyte vesicles may contain N-acetylgalactosamine and Ca^2+^ [93,131] while others have hypothesized that they contain lipid [33,132]. The density and size of these vesicles are reported to reduce and to become more centrally located during maturation of pig, cow and sheep oocytes [33,38,67,118,133]. This suggests that the contents of these vesicles may be important for oocyte maturation and subsequent embryo development.

### 5.1. Vesicle Quantity

Vesicle volume was greater in adult ewe oocytes compared to lamb oocytes prior to maturation. During maturation, vesicle volume decreased in both groups to reach a similar volume [33]. This decrease in volume was due to a decrease in the size of these organelles and suggests the more competent adult oocytes utilized more vesicle content than the less competent lamb oocytes. Following IVM, vesicle density was greater in denuded oocytes, which have a reduced developmental competence than cumulus enclosed oocytes, and both were lower than the even less competent immature oocytes [118]. These two studies indicate that either the quantity of vesicles prior to maturation, or the ability of the oocyte to metabolize the contents of these vesicles during maturation, contributes to oocyte competence.

It is interesting to note that vesicle density in an electron microscope study of sheep oocytes decreased but lipid droplet density did not [33]. Given the decrease in Nile red staining observed during maturation [118], this dye may be staining lipid other than that located in the electron-dense lipid droplets, particularly given that this dye can stain both saturated and unsaturated lipid [122]. This supports the notion that the vesicles observed by electron microscopy also contain lipid [33].

### 5.2. Vesicle Distribution

Vesicles were predominantly located in the center of both adult ewe and lamb oocytes, but due to their high volume density, occupied all but the periphery of the oocyte [33]. In pig oocytes, the distribution of the vesicles was described as even or central in prepubertal oocytes and central in postpubertal oocytes before IVM. After IVM, vesicles re-distribute to cortical or central (prepubertal) and cortical (postpubertal) locations [68].

Until the contents of these vesicles are confirmed it will be difficult to determine their role in developmental competence. The evidence so far is that these organelles contain a mixture of metabolites necessary for oocyte maturation and early embryo development and increased utilization of vesicles during maturation may be associated with improved oocyte quality.

## 6. Role of Cortical Granules in Oocyte Quality

Successful fertilization requires that mammalian oocytes have a defense mechanism to prevent polyspermy; that is, the penetration of the oocyte by more than one sperm [134]. One method for preventing polyspermy involves the biochemical modification of the zona pellucida (ZP) following the exocytosis of cortical granules. Cortical granules are secretory organelles produced from Golgi complexes during oocyte growth that become located close to the oocyte membrane as it matures [38,75,134,135]. When the sperm plasma membrane fuses with the oolemma, a series of intracellular signaling pathways are activated that stimulate exocytosis of the cortical granules releasing their contents [136]. The calcium chelator, BAPTA, has been shown to inhibit this process while calcium ionophore induces the release of cortical granules. Therefore, cytoplasmic calcium elevation appears to be required for this reaction [137,138]. The exact composition of cortical granules is difficult to determine due to the small amount of cortical granule material in mammalian oocytes but they have been shown to be rich in carbohydrates and contain a trypsin-like proteinase, ovoperoxidase, N- acetylglucosaminidase and several other proteins [134].

### Cortical Granule Quantity and Distribution

Cortical granules are located in clusters throughout the immature oocyte cytoplasm in cattle and sheep. However, in pig, mouse and human oocytes the cortical granules are never clustered and are located in the periphery of the oocyte. In all species, the cortical granules relocate to individual positions immediately below the oolemma during maturation [38,76,99]. O’Brien et al. [35] reported a lower volume fraction and size of cortical granules in prepubertal lamb oocytes compared to adult oocytes following IVM which may be related to the increased rate of polyspermy reported in oocytes from juvenile animals. However, there was no difference reported in the numerical volume density of cortical granules between juvenile and adult cow oocytes [93]. The redistribution of cortical granules to the oolemma in prepubertal calf and pig oocytes was delayed [51,68], but appeared to be normal in prepubertal lamb oocytes [33]. Prepubertal goat oocytes were described as having more electron dense and compact cortical granules than their adult counterparts but there appeared to be no difference in the distribution and migration of cortical granules between the age groups [139]. There appears to be some evidence that the size of the cortical granules and the time taken to relocate to the oolemma during maturation influences oocyte competence. However, further research is needed to better understand what regulates the size, composition and distribution of cortical granules in oocytes.

## 7. Manipulating Oocyte Organelles to Improve Quality

It is clear that the quantity of oocyte organelles affects oocyte quality, but very little research has been published on the manipulation of organelle quantity to improve oocyte competence. Cytoplasmic transfer, where a portion of cytoplasm from donor oocytes is microinjected into the recipient oocyte, has resulted in live-births in women with repeated implantation failure after assisted reproduction [140]. Whether or how the physiology of the resulting embryo is actually altered is not known. It has been presumed that the “cytoplasmic rescue” occurs as a result of additional mitochondria transferred to the recipient oocyte but few studies have confirmed this and other cytoplasmic factors may be responsible. El Shourbagy et al. [31] transferred purified preparations of mitochondria from BCB+ pig oocytes to maternally related BCB− oocytes and demonstrated an increase in fertilization rate, but later embryo development was not reported. Mice with an age-related decline in fertility have been shown to have mitochondrial dysfunction. Supplementation of old mice with coenzyme Q10 resulted in an increased ovarian reserve, restored mitochondrial respiration, membrane potential and ATP production to levels equivalent to young mice, and reduced chromosomal misalignment and spindle defects [141]. This demonstrates that manipulation of mitochondrial function can improve oocyte quality. However, whether coenzyme Q10 treatment altered mitochondrial numbers is not known. Recent research has identified other natural compounds, such as resveratrol, L-carnitine and lipoic acid, that can alter the mitochondrial and lipid content of in vivo and in vitro treated cells. Resveratrol can increase the expression of genes involved in mitochondrial biogenesis in some cell types [142,143] and has been shown to improve cleavage and blastocyst rates in pig and cow oocytes [144,145]. However, it is not known whether resveratrol altered mitochondrial number or function in those oocyte studies. L-carnitine is involved in the transportation of fatty acids into mitochondria and has been shown to improve mouse, pig, cow and sheep oocyte competence and to alter the distribution of oocyte mitochondria and lipid droplets [86,146,147,148,149]. These and other compounds that can increase mitochondria and lipid volume in cells should be explored further to determine if their use improves in vitro and/or in vivo oocyte quality. If successful, new supplements could be developed that can enhance both natural fertility and assisted reproduction across species.

## 8. Conclusions and Future Research

In summary, the research highlighted in this review article indicates that, by the end of maturation, oocytes with a greater volume or number of evenly distributed mitochondria, lipid droplets and vesicles, and cortical granules located immediately below the oolemma, have greater developmental competence (Table 1). However, there is a lack of good quantitative data using appropriate models of oocyte competence to understand the contribution lipids and other stored metabolites provide to improve oocyte quality.

Mitochondrial number is obviously critical to oocyte quality but the relationship between number, activity and mtDNA copy number needs to be further explored. Research into the association between morphological changes occurring in oocyte mitochondria during maturation and changes in their metabolic function is also needed. This, along with a more detailed knowledge of the composition of the lipid droplets and vesicles may lead to better understanding of the metabolic requirements of a good quality oocyte. Improved methods for the quantitative analysis of organelles and metabolites, such as MALDI-TOF mass spectrometry, desorption electrospray ionization (DESI) mass spectrometry, spinning disk confocal microscopy and serial block-face scanning electron microscopy, are available and in some cases, have been applied to single oocytes [150,151,152,153]. These methods will hopefully lead to a better understanding of the factors essential for oocyte quality, ways to identify good quality oocytes and improvements to oocyte developmental competence in vitro and in vivo.

## Figures and Tables

**Table 1:** Summary of organelle features in good and poor quality mature oocytes.

Organelle	Good Quality	Poor Quality
mito morphology	lighter matrix; hooded	denser matrix; vacuoles and granules; altered timing of hooded form
mito distribution	Even	Peripheral
mito number	Higher	Lower
mito activity	?	?
lipid volume	Greater	Smaller
lipid distribution	Even	Peripheral
vesicle volume	Greater	Smaller
CG distribution	Peripheral	clustered, even

mito, mitochondria; CG, cortical granules; ?, unknown.
